# Yeast Functional Genomic Screens Lead to Identification of a Role for a Bacterial Effector in Innate Immunity Regulation

**DOI:** 10.1371/journal.ppat.0030021

**Published:** 2007-02-16

**Authors:** Roger W Kramer, Naomi L Slagowski, Ngozi A Eze, Kara S Giddings, Monica F Morrison, Keri A Siggers, Michael N Starnbach, Cammie F Lesser

**Affiliations:** 1 Department of Medicine, Division of Infectious Diseases, Massachusetts General Hospital, Harvard Medical School, Cambridge, Massachusetts, United States of America; 2 Department of Microbiology and Molecular Genetics, Harvard Medical School, Boston, Massachusetts, United States of America; Tufts University School of Medicine, United States of America

## Abstract

Numerous bacterial pathogens manipulate host cell processes to promote infection and ultimately cause disease through the action of proteins that they directly inject into host cells. Identification of the targets and molecular mechanisms of action used by these bacterial effector proteins is critical to understanding pathogenesis. We have developed a systems biological approach using the yeast Saccharomyces cerevisiae that can expedite the identification of cellular processes targeted by bacterial effector proteins. We systematically screened the viable yeast haploid deletion strain collection for mutants hypersensitive to expression of the *Shigella* type III effector OspF. Statistical data mining of the results identified several cellular processes, including cell wall biogenesis, which when impaired by a deletion caused yeast to be hypersensitive to OspF expression. Microarray experiments revealed that OspF expression resulted in reversed regulation of genes regulated by the yeast cell wall integrity pathway. The yeast cell wall integrity pathway is a highly conserved mitogen-activated protein kinase (MAPK) signaling pathway, normally activated in response to cell wall perturbations. Together these results led us to hypothesize and subsequently demonstrate that OspF inhibited both yeast and mammalian MAPK signaling cascades. Furthermore, inhibition of MAPK signaling by OspF is associated with attenuation of the host innate immune response to *Shigella* infection in a mouse model. These studies demonstrate how yeast systems biology can facilitate functional characterization of pathogenic bacterial effector proteins.

## Author Summary

Many bacterial pathogens use specialized secretion systems to deliver effector proteins directly into host cells. The effector proteins mediate the subversion or inhibition of host cell processes to promote survival of the pathogens. Although these proteins are critical elements of pathogenesis, relatively few are well characterized. They often lack significant homology to proteins of known function, and they present special challenges, biological and practical, to study in vivo. For example, their functions often appear to be redundant or synergistic, and the organisms that produce them can be dangerous or difficult to culture, requiring special facilities. The yeast Saccharomyces cerevisiae has recently emerged as a model system to both identify and functionally characterize effector proteins. This work describes how genome-wide phenotypic screens and mRNA profiling of yeast expressing the *Shigella* effector OspF led to the discovery that OspF inhibits mitogen-activated protein kinase signaling in both yeast and mammalian cells. This inhibition of mitogen-activated protein kinase signaling is associated with attenuation of the host innate immune response. This study demonstrates how yeast functional genomic studies can contribute to the understanding of pathogenic effector proteins.

## Introduction

Bacterial pathogens have evolved numerous mechanisms to evade host cell defenses and promote infection. One strategy common to many pathogens is the manipulation of host cell processes by bacterial effector proteins that are directly delivered into host cells by specialized secretion systems [1]. Although these effector proteins are critical to pathogenesis, relatively few are well characterized. A number of effectors whose functions are understood manifest activity in Saccharomyces cerevisiae analogous to their activity in mammalian cells during infection, presumably because these proteins target fundamental cellular processes conserved among all eukaryotes. Consequently, we and others have exploited yeast as a model organism in which to identify and characterize bacterial effector proteins [2–5].

When expressed in yeast, bacterial effectors that target conserved eukaryotic cellular processes often inhibit growth [2,3,6] and/or exhibit conserved subcellular localization patterns [3,7]. Growth inhibition provides a measurable phenotype that can indicate the degree of disruption of cellular processes [8,9]. Because over 75% of the yeast genome is functionally annotated, systematic identification of genes or proteins that modulate a phenotype can identify pathways and processes involved in that phenotype [9–13], which, in turn, can provide insights into its etiology. Thus, by systematically screening for yeast deletion strains hypersensitive to expression of a bacterial effector protein, cellular processes that buffer yeast against the toxicity of the effector can be identified. As proof of principal, we applied this approach to OspF, a *Shigella* effector protein.

OspF is found in all pathogenic *Shigella* species [14] and is an established substrate of the Shigella flexneri type III secretion system [15]. At the start of this study, nothing was known about its molecular activity or role in pathogenesis. Having observed its mild toxicity to wild-type yeast under select conditions, we screened for yeast deletion strains hypersensitive to OspF expression. In parallel, we measured alterations in mRNA levels in wild-type yeast expressing OspF. The resulting complementary views of OspF activity led us to hypothesize, and subsequently demonstrate, that OspF inhibits both yeast and mammalian mitogen-activated protein kinase (MAPK) signaling cascades. Furthermore, this alteration in signaling is associated with attenuation of the host innate immune response to *Shigella* infection in the mouse lung model. These results are consistent with those of Aribe and colleagues who recently determined that OspF is a MAPK phosphatase [16]. Our study demonstrates how systems biological approaches using yeast can generate testable hypotheses regarding the roles of bacterial effector proteins in pathogenesis.

## Results

### A Genome-Wide Phenotypic Screen Identified Yeast Deletion Strains Hypersensitive to OspF Expression

**Figure 1:**
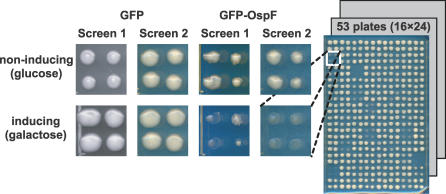
Yeast Growth Inhibition Screen The entire yeast haploid deletion strain collection was transformed four separate times, twice with a plasmid that conditionally expresses GFP and twice with a plasmid that conditionally expresses GFP-OspF. Transformants were spotted in quadruplicate onto solid media trays such that up to four biological replicas were examined in each screen. Each screen was conducted in duplicate. Images shown reflect growth after 48 h.

OspF is one of several *Shigella* effectors that inhibit growth when expressed in yeast (Figure S1 and Protocol S1). OspF also exhibits conserved localization to both the cytoplasm and nucleus when expressed de novo in yeast and mammalian cells (Figure S2). While OspF is toxic to yeast grown in synthetic liquid media, fusion of OspF to green fluorescent protein (GFP) partially relieves its toxicity, and growth in rich media almost completely abolishes its toxicity. We hypothesized that OspF activity is independent of the growth medium and that yeast compensatory mechanisms are merely enhanced in rich media. To identify genes that contribute to these mechanisms, we systematically screened the yeast haploid deletion strain collection for null alleles specifically impaired by expression of GFP-OspF in rich media (Figure 1, Protocols S2 and S3).

Each strain in the deletion collection contains a complete deletion of one nonessential open reading frame (ORF). The deletion collection includes 4,773 strains covering 77% of the ∼6,200 annotated yeast ORFs. While most of these deletion strains grow as well as wild-type when cultured in rich media, growth of 98 of the deletion strains was severely and reproducibly impaired by expression of GFP-OspF but not of GFP alone (Figure S3). Fifteen of the deletion strains were excluded from further analyses. These included two strains deleted for dubious ORFs, three whose gene products are required for galactose metabolism (our inducing condition), and ten whose gene products are ribosomal structural components. Thus, our result set contained 83 genes hypersensitive to OspF (Table S1).

Our screen for null alleles hypersensitive to OspF is analogous to screens for synthetic lethal (SL) interactions. Rather than inferring functional relationships between genes by identifying pairs of mutant alleles that when combined result in inviability, we sought to infer functional relationships between yeast genes and OspF toxicity by identifying null alleles that exacerbate OspF toxicity. Null alleles that are hypersensitive to OspF expression effectively define SL interactions with OspF. No consensus exists, however, regarding proper interpretation of SL interactions in terms of pathways, and we faced a similar interpretive dilemma with the 83-gene result set of our hypersensitivity screen.

Two interpretations motivated by the “circuit” concept of a pathway have currency. One explains SL interactions as resulting from cumulative insults to the same pathway (a “serial” circuit) that reduce its efficiency below some critical threshold. Alternatively, synthetic lethality may result from simultaneous insults to mutually compensating or “buffering” processes (“parallel” circuits). Clearly, these interpretations depend on what constitutes a pathway, a concept which itself remains loosely defined. Nonetheless, these interpretations of SL interactions serve to delineate a range of possible explanations for hypersensitivity to OspF. Specifically, the result set of OspF-hypersensitive deletion strains might be enriched in proteins involved in cellular pathways directly insulted by OspF (by analogy with the “serial” circuit interpretation). Alternatively, it might be enriched in pathways that mitigate the effects of OspF expression (by analogy with the “parallel” circuit interpretation). In either case, it was essential to identify cellular processes represented in the result set.

### Enrichment of Biological Processes in OspF Hypersensitive Null Alleles

To identify pathways impaired among the 83 hypersensitive strains, we used the online data-mining tool FuncAssociate [17] to identify gene ontologies enriched among the deletion strains. Gene ontologies are semi-hierarchical classifications of genes based on the roles their proteins play in biological processes (process ontologies), their cellular localizations (component ontologies), and their biochemical activities (molecular function ontologies). One of the major advantages of conducting genome-wide studies in yeast is the wealth of information available regarding individual yeast gene products. For example, over 75% of the yeast genome is functionally annotated to at least one type of gene ontology [18].

**Figure 2:**
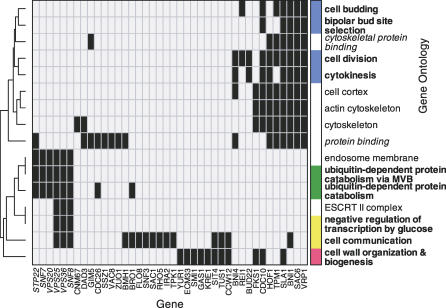
Ontologies Enriched among the 83 Genes That Are Essential in Yeast Expressing OspF A black cell indicates membership of its column's gene in its row's ontology. Two independent types of hierarchy are represented in the matrix. Columns and rows have been hierarchically clustered and the dendrogram on the left shows the results of row clustering (column dendrogram not shown). Gene ontologies are hierarchical classifications, and the color bands indicate hierarchically related groups of process ontologies (in bold type). Ontologies in italics are molecular functions; the remaining five are components.

FuncAssociate revealed that 25 ontologies represented by the genes deleted in 79 hypersensitive strains were significantly overrepresented (theoretical *p*-values < 0.001) (Table S2). We simplified these results by eliminating redundant and relatively broad ontologies such as “cellular process,” “cytoplasm,” and “cell organization and biogenesis” because these ontologies (near the roots of the hierarchies) are shared by hundreds or thousands of genes (Table S2). Additionally, in the six cases where two hierarchically related ontologies included the exact same set of genes in our results, the more general (less descriptive) ontology was discarded (Table S2). In order to visualize relationships among the remaining 16 ontologies, we performed two-dimensional clustering of the ontologies and the genes they subsume (Figure 2). Of the remaining 16 ontologies, nine were biological process ontologies. With respect to hierarchical relationships, these process ontologies fell into four groups rooted in “cell communication,” “cell wall organization and biogenesis,” “ubiquitin-dependent protein catabolism,” and “cell division.”

### OspF Is Congruent to Genes Impaired in Cell Wall Biogenesis

For another perspective, we carried out a second type of enrichment analysis using existing SL interaction data. “Congruent” gene pairs have been defined as those sharing SL interaction partners [19]. Again by analogy, we defined a gene to be congruent to OspF expression if its set of SL interaction partners has statistically significant overlap with mutant alleles hypersensitive to OspF (Figure S4). Intuitively, if OspF is congruent to a gene, then expression of OspF effectively mimics mutation of that gene. We scanned the available database of 9,019 SL interactions encompassing 2,286 ORFs [18] for genes congruent to OspF. Almost all of the genes that are congruent to OspF encode proteins involved in either the cell wall integrity (CWI) pathway or chitin biosynthesis, both of which are related to cell wall biogenesis (Table 1) [20].

**Table 1:**
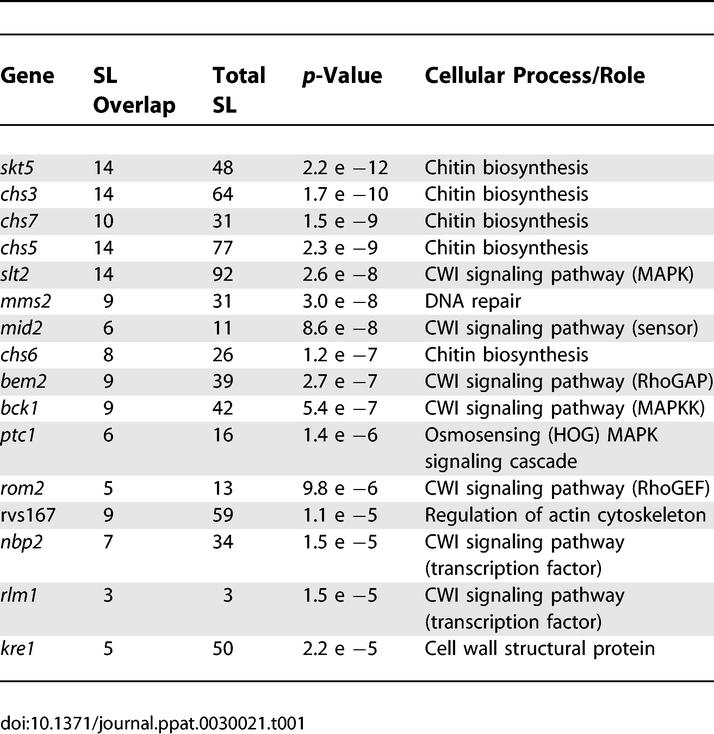
Congruence between OspF and Mutant Alleles of Designated Genes

The CWI pathway regulates cell wall biogenesis during the cell cycle as well as under conditions that perturb the cell wall. All of the proteins involved in chitin biosynthesis in Table 1 either directly encode or regulate the activity of chitin synthase III, a protein responsible for synthesis of the chitin ring at the bud neck during cell division and for chitin in the lateral cell wall. Although chitin normally accounts for only 1%–2% of the yeast cell wall, chitin can contribute up to 20% of the cell wall under times of stress [21]. Furthermore, with the exception of *KRE1,* strains deleted for none of the genes congruent to OspF were hypersensitive to OspF, suggesting that these genes represented targeted rather than compensating pathways. Thus, the results of this second analysis raised the possibility that OspF acts to inhibit the CWI pathway and/or chitin biosynthesis.

The implication of cell wall integrity in OspF toxicity by two independent analyses focused our attention on the yeast cell wall. Furthermore, the fact that the CWI pathway is a highly conserved MAPK signaling cascade made it a plausible target (in yeast) for an effector protein from a mammalian pathogen. Although this hypothesis resulted from data mining two different types of systematic data, functional annotation and genetic interaction data, it was based on one type of experimental data: identification of genes that modulate OspF toxicity. For a complementary systems view we also identified genes whose expression was modulated by OspF.

### OspF Alters mRNA Levels of Genes Regulated by the CWI Pathway

We examined the transcriptional response of wild-type yeast to OspF expression using Affymetrix Yeast GeneChips. mRNA profiling studies conducted in triplicate identified only 19 genes regulated greater than 2-fold (Table 2) 3 h after the induction of OspF expression. Strikingly, the second-most downregulated gene in all three independent mRNA profiling experiments encodes CWP1, a major structural component of the yeast cell wall. Although loss of this protein does not result in measurable alterations in yeast growth under laboratory conditions, *Δcwp1* strains are sensitive to agents that impair CWI, and this gene is normally upregulated in response to cell wall stress [22]. Furthermore, CWP1 is one of ∼20 proteins whose expression is regulated by the CWI pathway [23]. Activation of the CWI pathway results in phosphorylation of RLM1, a transcription factor that induces expression of 19 genes including *CWP1* and *PRM5* and represses expression of five genes, including *FIT2* [23]. The OspF-dependent reversed regulation of *CWPI, PRM5,* and *FIT2* suggested that OspF expression directly or indirectly inhibits RLM1-regulated transcription.

**Table 2:**
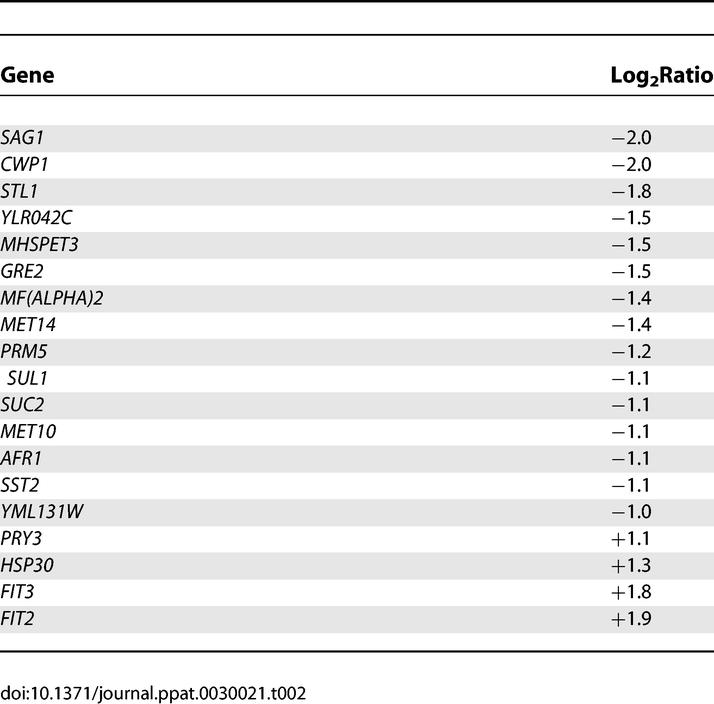
Genes Differentially Regulated in Response to OspF Expression

Since we examined alterations in mRNA expression patterns among unsynchronized cells and the CWI pathway is only periodically induced during the cell cycle, it was perhaps not surprising that more significant alterations were not observed in additional RLM1-regulated genes. Indeed, when grown under normal laboratory conditions, activation of the CWI pathway is undetectable in asynchronous cells [22]. Nevertheless, although expression of the remaining RLM1-regulated genes was not altered greater than 2-fold, in general those genes normally induced by RLM1 were repressed in the presence of OspF, and those genes normally repressed were induced 3 h after the induction of expression of OspF (Figure S5).

### OspF Inhibits Signaling of the CWI Pathway

**Figure 3:**
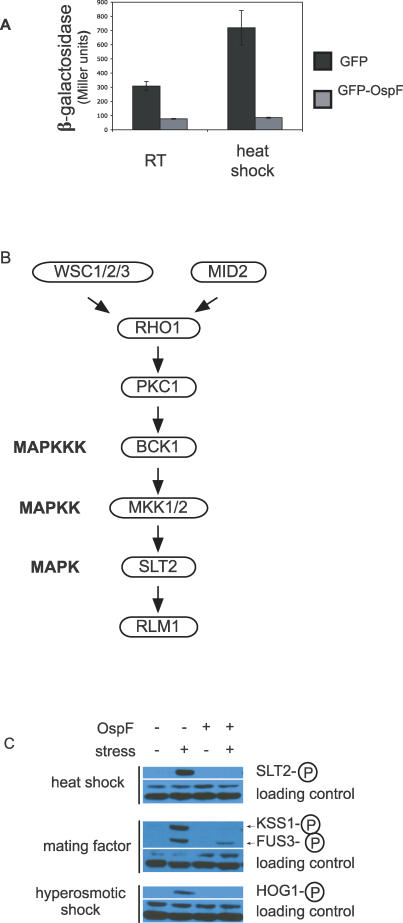
OspF Inhibits the Yeast CWI Pathway by Inhibition of MAPK Phosphorylation (A) Summary of the activity of an RLM1-regulated β-galactosidase reporter in response to heat shock in the presence or absence of OspF. (B) Outline of the CWI pathway. (C) Yeast containing empty vector or a plasmid that conditionally expresses OspF were subjected to the designated stress 2 h after the induction of expression of OspF (see Materials and Methods). Representative immunoblots used to assay for activation of each of four MAPK signaling pathways: SLT2 (CWI pathway), FUS3 (mating pathway), KSS1 (invasive growth pathway), and HOG1 (high-osmolarity glycerol pathway) are shown. The circled P denotes phosphorylated versions of the proteins. The blots were probed with the anti-PSTAIRE antibody that recognizes CDC28 and PHO85 as a loading control. Each experiment was conducted at least in triplicate with similar results.

We next investigated whether OspF expression does indeed regulate expression of the CWI pathway by monitoring the effects of GFP-OspF expression on an RLM1 transcriptional reporter. This well-characterized reporter contains two RLM1 binding sites fused to the minimal *CYC1* promoter driving *lacZ* [24]*.* Expression of this reporter gene is detectable in asynchronous yeast grown under standard laboratory conditions and responds appropriately to perturbations that activate the CWI pathway [3,10,25]. We observed that GFP-OspF inhibits basal levels of expression of this CWI pathway reporter and inhibits activation of the CWI pathway in response to heat shock (Figure 3A). Thus, expression of OspF appears to inhibit activation of the CWI pathway.

### OspF Inhibits Yeast MAPK Phosphorylation

Many of the components of the yeast CWI pathway are highly conserved among eukaryotes (Figure 3B). In fact, many bacterial effector proteins, including *Shigella* IpgB2 and *Yersinia* YopE, have been demonstrated to inhibit specific steps in this pathway in both yeast and mammalian cells [3,10,25]. IpgB2 is a Rho mimic that activates RHO signaling while YopE is a RhoGAP. In order to determine where OspF targets the CWI pathway in relationship to Rho1, we first screened for alterations in the integrity or polarity of the yeast cytoskeleton in response to expression of OspF. No perturbations were observed (unpublished data) suggesting that OspF targets a component of the CWI signaling pathway downstream of Rho1.

To further localize the action of OspF, we took advantage of the cross-reactivity of the mammalian phospho-specific p42/44 MAPK antibodies and phosphorylated Slt2. Since CWI pathway activation is barely detectable in wild-type yeast grown under standard laboratory conditions, we assayed for the ability of OspF to block activation of the MAPK pathway by either heat or hypoosmotic shock. OspF inhibited phosphorylation of SLT2 in response to both of these conditions, but did not alter total SLT2 levels (Figure 3C, unpublished data). Thus, OspF targets a protein upstream of RLM1 and downstream of RHO1 in the CWI pathway.

### OspF Inhibits Phosphorylation of Additional Yeast MAPKs

Several lines of evidence suggested that OspF might target additional cellular processes. For example, the growth inhibition of wild-type yeast due to expression of OspF cannot be explained by inhibition of just the CWI pathway since deletion strains that no longer express essential components of the CWI pathway are not inhibited for growth under the same conditions. Yeast encode five additional MAPK signaling cascades including the mating pathway (Fus3), the invasive growth pathway (Kss1), the hyperosmotic growth (HOG) pathway, the sporulation pathway (Smk1), and Mlp1, a second MAPK implicated in the CWI pathway [26]. A strain deleted in all six MAPKs has been reported to be impaired for growth [27]. The severe growth inhibition of yeast observed in liquid media could be accounted for by OspF inhibiting multiple yeast MAPK signaling pathways. Indeed, we observed that OspF expression also impaired phosphorylation of three additional yeast MAPKs: Hog1, Kss1, and Fus3 (Figure 3C). Thus, OspF appears to nonspecifically inhibit phosphorylation of yeast MAPKs. This general inhibition of yeast MAPK signaling pathways likely explains the OspF hypersensitivity of deletion strains not directly related to cell wall biogenesis. Because MAPK cascades are highly conserved among all eukaryotes, and OspF is a virulence protein from a human pathogen, it seemed likely that OspF would likewise target mammalian MAPK pathways.

### OspF Inhibits Mammalian MAPK Signaling Pathway

**Figure 4:**
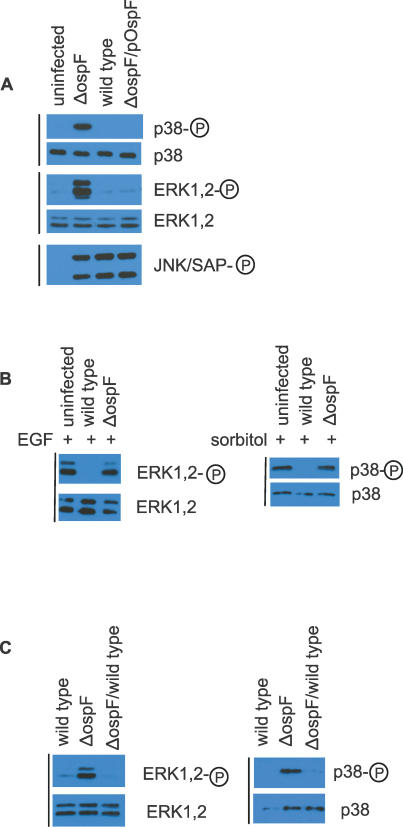
OspF Inhibits ERK and p38 Phosphorylation (A) Immunoblots of extracts of HeLa cells infected with wild-type, *ΔospF,* or *ΔospF/*pOspF *Shigella* for 1 h. (B) pOspF indicates the OspF complementing plasmid. Cell lysates were probed with the designated antibodies. Immunoblots of extracts of uninfected HeLa cells or HeLa infected with wild-type or *ΔospF Shigella* for 1 h and then exposed to 50 ng/ml EGF or 0.4 M sorbitol to activate ERK and p38 signaling, respectively. (C) Immunoblots of extracts of HeLa cells infected with wild-type *Shigella* (MOI 10:1), *ΔospF Shigella* (MOI 10:1), or mix of wild-type *Shigella* (MOI 5:1) plus *ΔospF Shigella* (MOI 5:1) for 1 h. In all cases, each experiment was conducted at least in triplicate with similar results.

To test whether the presence of OspF altered MAPK signaling during infection, we compared MAPK phosphorylation patterns in mammalian cells infected with wild-type or *ΔospF Shigella.* As previously observed, infection with wild-type *Shigella* resulted in strong SAP/JNK phosphorylation [28], weak ERK phosphorylation [29], and no detectable p38 phosphorylation (Figure 4A). In contrast, infection with *ΔospF Shigella* resulted in robust phosphorylation of all three MAP kinases, without alteration of the overall levels of MAP kinases in the cells. The *ΔospF Shigella* phenotype was complemented by expression of OspF from its endogenous promoter on a low copy-number plasmid (Figure 4A). These observations suggested that the presence of OspF either inhibits ERK and p38 phosphorylation or the absence of OspF stimulates their phosphorylation.

The observations in yeast suggested that the presence of OspF is sufficient to inhibit MAPK phosphorylation. Several experiments were conducted to test whether this is the case in mammalian cells. First, we compared the ability of wild-type and *ΔospF Shigella* to inhibit MAPK phosphorylation in response to the addition of exogenous agents that induce MAPK signaling. Wild-type, but not *ΔospF Shigella,* inhibit activation of ERK phosphorylation in response to epidermal growth factor and p38 phosphorylation in response to sorbitol (Figure 4B). Second, we coinfected mammalian cells with wild-type and *ΔospF Shigella* at a ratio of 1:1. The *ΔospF Shigella* strain demonstrated no defect in invasion alone or in competition with wild-type *Shigella* (unpublished data). The presence of OspF in the mixed infection was sufficient to inhibit MAPK signaling pathways (Figure 4C). Thus, OspF inhibits MAPK phosphorylation in both yeast and mammalian cells. While this inhibition is nonspecific in yeast, the signaling of the mammalian SAP/JNK pathway appeared to be unaffected by the presence of OspF.

### OspF Inhibits MAPK Downstream of the MAPKKK

**Figure 5:**
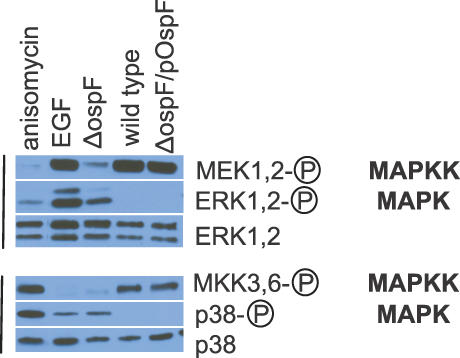
OspF Stimulates MAPKK Activation and Inhibits MAPK Activation Immunoblots of extracts of HeLa cells infected with wild-type, *ΔospF,* or *ΔospF/*pOspF *Shigella* or exposed to anisomycin or EGF, to activate p38 and ERK signaling, respectively. The latter two reagents were added as positive controls for detection of activation of phosphorylated MAPK and MAPKK. Cell lysates were probed with the designated antibodies. Each experiment was conducted at least in triplicate with similar results.

Inhibition of MAPK phosphorylation is a strategy used by other bacterial pathogens, including *Yersinia* species and *Bacillus anthracis,* to modulate the innate immune response. The *Yersinia* type III effector YopJ is an acetyltransferase that inhibits MAPKK phosphorylation by direct modification of residues that are normally phosphorylated [30]. B. anthracis lethal factor, a metalloprotease, cleaves the amino-termini of MAPKKs [31]. To determine whether *Shigella* also inhibits MAPK phosphorylation by modulation of MAPKKs, we monitored the phosphorylation state of the MAPKKs directly upstream of ERK and p38. In both cases, infection with wild-type, but not *ΔospF Shigella,* resulted in the accumulation of phosphorylated MAPKKs (Figure 5) to levels markedly greater than those observed in the absence of OspF. The presence of full-length phosphorylated MAPKKs in the absence of phosphorylated MAPKs suggests that OspF inhibits MAPK phosphorylation by a novel mechanism, by either blocking phosphorylation of MAPKs by activated MAPKKs or by dephosphorylating activated MAPKs. These results are consistent with recent observations that OspF is a MAPK phosphatase for ERK and p38 [16].

### OspF Attenuates the Host Innate Immune Response to *Shigella* Infection

Mammalian MAPK signaling pathways regulate diverse cellular activities including cell proliferation, differentiation, motility, survival, and innate immunity. The innate immune response is activated when host cells recognize pathogen-associated molecular patterns that include microbial products like peptidoglycan and lipopolysaccharide. The pathogen-associated molecular patterns are recognized by pathogen recognition receptors, like the extracellular Toll-like receptors and the intracellular nucleotide-binding oligomerization domain proteins (for review see [32]). For example, after *Shigella* invade host cells, mammalian NOD1 binds the bacterial peptidoglycan, resulting in activation of the SAP/JNK and ERK MAPK signaling pathways as well as NF-κB activation [28]. Many pathogens, including *Shigella,* modulate this host immune response presumably to promote their own survival. Specifically, the *Shigella* effectors OspG and IpaH9.8 downregulate the innate immune response by inhibition of NF-κB activation [33,34]. Therefore, we hypothesized that OspF inhibition of MAPK signaling might also serve to downregulate the mammalian immune response.

**Figure 6:**
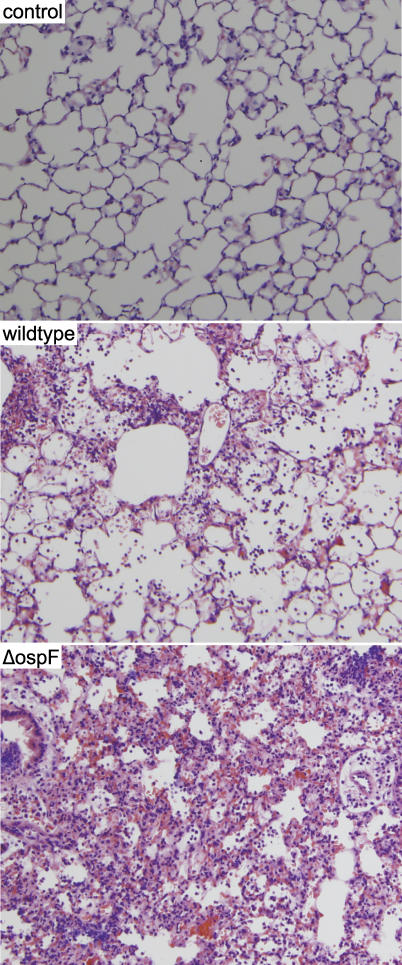
OspF Is Associated with Attenuation of the Host Innate Immune Response The images shown are of hematoxylin- and eosin-stained sections of lungs of mice 24 h after infection with wild-type or *ΔospF Shigella* or injection with equivalent volume of phosphate buffered saline.

We next investigated whether downregulation of MAPK signaling pathways by *Shigella* in cell culture reflected in vivo alterations in the innate immune response to infection. Although *Shigella* infections are normally restricted to the intestines in humans, the bacterium is unable to sustain an infection in the intestines of adult mice. However, the mouse lung infection is an established model for monitoring the immune response to *Shigella* infections [35]. After determining that *ΔospF Shigella* were not defective in invasion of host cells or in cell-to-cell spread (unpublished data), we investigated the innate immune response to *Shigella* in the mouse lung infection model. As expected, 24 h after infection the lungs of mice infected with wild-type *Shigella* showed an inflammatory infiltrate dominated by polymorphic neutrophils (Figure 6). In contrast, infection with *ΔospF Shigella* triggered a markedly more aggressive immune response as manifested by the increase in polymorphic neutrophils in the lungs. Furthermore, the visible edema and hemorrhage resulted in considerable destruction of the lung architecture (Figure 6). These findings suggest that the presence of OspF attenuates the host innate immune response.

## Discussion

Although unicellular eukaryotes such as yeast cannot serve as models for the bacterial infection of host cells, the utility of S. cerevisiae for investigating the molecular activities of effector proteins is well established. Until now, little has been done to exploit the genetic tractability of yeast to determine cellular targets of effector proteins. In this study, we present the first use of yeast systems biology to identify a function for a poorly understood bacterial effector protein. By integrating complementary systems-level snapshots of the cellular perturbation caused by OspF, we were able to determine that OspF targets a well-characterized yeast-signaling pathway. These observations led us to hypothesize and subsequently demonstrate that OspF inhibits highly conserved MAPK signaling pathways including those that regulate cell wall biogenesis in yeast and the host innate immune response in mammals. These results are in agreement with the recent demonstration that OspF is a MAPK phosphatase specific for ERK and p38 [16]. Although, there is also evidence that OspF can activate ERK phosphorylation in polarized epithelial cells [36].

One of the hallmarks of *Shigella* infections is the dramatic inflammatory response characterized by the recruitment of polymorphic neutrophils to sites of infection. This response facilitates access of *Shigella* to the basolateral surface of epithelial cells where this intracellular pathogen mediates its own uptake into cells via a type III secretion system. *Shigella* trigger activation of the innate immune response, at least in part, through the action of pathogen-associated molecular patterns like LPS [37] and peptidoglycan [28]. *Shigella* also down regulate the innate immune response by the action of several proteins including OspF. The *Shigella* effector proteins, OspG and IpaH9.8, inhibit NF-κB activity resulting in decreased production of proinflammatory cytokines [33,34]. In addition, the ShiA protein down regulates the innate T-cell response [38]. OspF-mediated downregulation of the innate immune response by inhibition of MAPK signaling complements the actions of these other *Shigella* proteins. These proteins presumably allow *Shigella* to fine-tune the host innate immune response over the course of infection.

OspF homologs are found in pathogens of both plants and animals including Salmonella typhimurium (SpvC) and Pseudomonas syringae (HopAI1) (Figure S6). HopAI1 was recently demonstrated to inhibit activation of the plant innate immune response by an unknown mechanism [39]. These observations suggest the existence of a new class of bacterial proteins, common to pathogens of both plants and animals that modulate the host innate immune response by inhibiting MAPK phosphorylation.

Our study demonstrates how genome-wide yeast screens can help generate testable hypotheses about the roles of bacterial effector proteins in pathogenesis. We applied a standard technique of statistical data mining, enrichment analysis, to integrate our screen results with two of the many types of systems data available for *S. cerevisiae,* genetic interactions and gene ontology annotations. This implicated CWI in OspF toxicity. The juxtaposition of this observation with the apparent reverse regulation of the CWI pathway in microarray experiments provided the crucial insight. Our approach was necessarily heuristic. For example, the set of genes exhibiting differential expression in response to OspF was of a size that one might dismiss as uninformative if it were the only set of data available, but the implication of CWI involvement by the hypersensitivity screens made the presence of any CWI pathway–regulated genes among those differentially regulated conspicuous.

Considerable evidence also motivated our focus on the cell wall. For example, at least five OspF hypersensitive deletion strains not accounted for in the enrichment analyses are impaired in protein mannosylation and glycosylphosphatidyl-inositol anchor biosynthesis. Both of these post-translational modifications are abundant among yeast cell wall proteins [40]. The corresponding ontologies were not statistically significant in our analyses, but their biological significance to cell wall biogenesis is well established. Similarly, six of the eight genes annotated to “ubiquitin-dependent protein catabolism” (italicized in Figure 2) are involved in regulation of an alternative cell wall biogenesis pathway which only becomes essential when the normal cell wall biogenesis pathway is perturbed [41,42]. These and other observations highlight the limitations of statistical methods and the importance of complementing formal analysis with an in-depth literature review to accurately interpret screen results.

The demonstrated inhibition of the CWI pathway by OspF accounts for much, but not all, of the observed hypersensitivity in deletion strains. The CWI pathway is constitutively active in at least three of the OspF hypersensitive cell wall biogenesis strains *(Δgas1, Δsmi1,* and *Δfks1)* [22]. Presumably this activation is essential for their survival since these deletion strains are synthetically lethal in combination with mutations that impair the CWI pathway *(Δslt2* and *Δbck1)* [11]. However, other pathways and processes were also implicated to which no clear CWI connection exists.

We assume that the multiplicity of process ontologies enriched in the hypersensitive strains reflects pathway dependencies centered on the cellular perturbation caused by OspF. For example, if a given pathway is required to mitigate toxicity of OspF, then any cellular process on which that pathway depends, will probably also be intolerant of impairment by gene deletion, though perhaps to a lesser degree. From this perspective, it is possible that OspF inhibition of cell wall biogenesis via the CWI pathway accounts for the hypersensitivity of cell division-associated null alleles since successful cell division depends strongly on CWI. Because yeast are under high osmotic pressure, budding and cytokinesis must be tightly coordinated with cell wall growth and remodeling to maintain CWI throughout the process. Defects in the cell wall can result in failure of cell division due to incomplete cytokinesis or cell lysis [43]. The congruence of chitin biosynthesis genes to OspF expression supports this speculation since chitin is a cell wall constituent involved in cell division [40]. However, concrete evidence explaining hypersensitivity of the cell division-associated null alleles in terms of OspF inhibition of the CWI pathway is lacking. The hypersensitivity of these null alleles may also be related to OspF inhibition of multiple MAPK cascades. The unanimity of our analyses in implicating CWI is likely due to the fact that this is the only MAPK cascade with demonstrated basal activity in laboratory conditions.

Our study demonstrates how genome-wide yeast screens can help generate testable hypotheses about the roles of bacterial effector proteins in pathogenesis. The multiple perspectives gained from two genome-wide experiments (as well as from multiple analyses of individual experiments) allowed us to effectively “triangulate” the process being perturbed by OspF, as well as to identify the nature of the perturbation. Other choices of screens, e.g., genome-wide identification of OspF binding targets using protein arrays or coimmunoprecipitation assays or identification of toxicity-suppressing conditions in synthetic liquid media (in which OspF is more toxic to yeast), would likely have resulted in a different path to the same discovery. For example, a preliminary genome-wide screen for suppressors of OspF toxicity in liquid media suggested that *Δsac7* suppresses OspF toxicity. SAC7 is a RhoGAP whose absence should lead to increased activation of Rho1, so it makes sense, given what we establish in this paper, that it would suppress OspF toxicity.

Genome-wide screens are potentially applicable to any bacterial effector that exhibits evidence of conserved activity in yeast such as toxicity or conserved localization. Hypersensitivity screens only make sense for effectors that exhibit mild or conditional toxicity, but other kinds of screens can be applied to extremely toxic effectors. For example, Alto and colleagues recently demonstrated how genome-wide suppressor screens of the yeast deletion strain collection can identify the cellular targets of effector proteins whose expression severely inhibits yeast growth [10]. They confirmed that *Shigella* IpgB2 mimics activated Rho1 in yeast when they observed that three deletion strains, all downstream components of the MAPK signaling cascade regulated by Rho1, were resistant to IpgB2 toxicity. In this case, since expression of IpgB2 activates a nonessential signaling pathway, they were able to isolate suppressors that blocked persistent activation of the pathway. However, if effector proteins are toxic because they directly target an essential cellular process, it may not be possible to isolate suppressors by screening yeast strains that contain null alleles of nonessential genes. Alternatively, suppressors might be found by screening for yeast genes whose overexpression suppresses toxicity. A genome-wide library of such constructs is now available [44].

In summary, this study exemplifies how contemporary analytic tools and the simplest of eukaryotes, *S. cerevisiae,* can contribute to the study of bacterial effectors. Even when bacterial proteins target cellular processes like innate immunity, which are not conserved among all eukaryotes, if elements of these processes like MAPK signaling cascades are conserved, then screens in yeast can be helpful in elucidating a function for the effector proteins. This systems approach should be generally applicable to numerous microbial virulence proteins. Moreover, since this approach only requires bacterial DNA, it should be particularly valuable for pathogens difficult to genetically manipulate or dangerous to culture.

## Materials and Methods

### Yeast plasmids and strains.

The gene encoding OspF was PCR-amplified from 2457T S. flexneri serotype 2a and subcloned into pFUS-GFP and pFUS-HIII1 [3]. Low-copy number versions of the GFP-OspF and GFP expression plasmids were constructed by homologous recombination in yeast to create pRS316-GAL10-GFP-OspF, pRS313-GAL10-GFP-OspF, pRS316-GAL10-GFP, and pRS313-GAL10-GFP. The *nat1* gene, which confers resistance to nourseothricin, was cloned into the pPR316-based plasmids to create pRS316-GAL10-GFP-OspF-CN and pRS316-GAL10-GFP-CN. Genes encoding S. typhimurium SpvC and P. syringae HopAI1 were PCR-amplified from genomic DNA preparations (E. Hohman and W. Songnuan, Massachusetts General Hospital ) and transferred into pBY011-D123 (B. Bhullar, Harvard Institute of Proteomics), a yeast expression plasmid using Gateway technology (Invitrogen, http://www.invitrogen.com).

### Bacterial plasmids and strains.

All infections were performed with S. flexneri 2457T serotype 2a. *ΔospF Shigella* was constructed using the λred recombinase-mediated recombination system [45].

A PCR fragment encompassing *ospF* plus 90 bp upstream and 300 bp downstream was cloned in pAM238 (pSC101 ori) to create pOspF, a complementing plasmid.

### Yeast transfers.

All yeast transfers in 96- or 384-well arrays were conducted using a Biorobot 3000 robot (Qiagen, http://www1.qiagen.com) outfitted with one of three floating pin tools described below (V&P Scientific, Incorporated, http://www.vp-scientific.com).

### Screen for deletion strains hypersensitive to GFP-OspF.

The entire yeast **MATα** haploid deletion strain collection (53 × 96-well plates/set) (Open Biosystems, http://www.openbiosystems.com) was transformed twice with pRS316-GAL10-GFP-OspF-CN and twice with pRS316-GAL10-GFP-CN using our 96-well transformation protocol (Protocol S2). Transformants were first selected under noninducing conditions (SC-URA 2% glucose) on solid media. Each transformation was plated in quadruplicate to create a 384-well array. Colonies were transferred to a second noninducing plate to “normalize” the yeast in each spot, since each individual yeast transformation varied in efficiency. The yeast from this plate were transferred to rich- (YEP) inducing (2% galactose) and noninducing (2% glucose) media. In the case of inducing media, clonNAT (70 μg/ml) was added to the media to ensure maintenance of the transformed plasmid. The plates were incubated at 30 °C for ∼48 h and then quantitated. Since the transformation efficiency was relatively poor, we assumed that each of the four transformation spots generated from each transformed strain was composed of independent transformations. Thus, each transformation resulted in four independent biological replicates. Each screen was repeated twice, thus we screened up to eight independent biological samples of each strain in the collection (Protocol S3, Figure S7, Tables S3 and S4).

### Comparison of OspF screen results with SL data.

We extracted the SL interactions from the exhaustive table of protein interaction data from *Saccharomyces* Genome Database (obtained by File Transfer Protocol, downloaded on 9 September 2006). We filtered this data to include only those SL interactions qualified as “inviable.” The bulk of this data comes from several large-scale screens, but over 1,000 small-scale screens also contribute to the total of 9,019 unique interactions between 2,286 genes. Only 1,041 of these interactions have been experimentally confirmed symmetric. For the purpose of our analysis we assumed all listed interactions were symmetric.

Of the 83 null alleles hypersensitive to OspF, 58 were included among the 2,286 genes in the SL database. To assess the significance of overlap between these 58 and the prey sets of each of the 2,286 genes, we calculated the chance of each intersection occurring in randomly selected samples as follows:

Let W be the set of all genes in the interaction database. Let H = ( x ∈ W: null alleles of x are hypersensitive to OspF }. That is, H is the OspF “hit” set. For each gene *g* in W let P(*g*) = ( x ∈ W/*g* : x is synthetically lethal with *g* }. P is the “prey” set of *g.* Then the probability of H∩P containing k or more genes is given by the hypergeometric distribution:

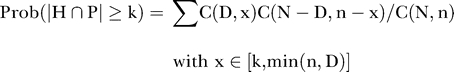
where

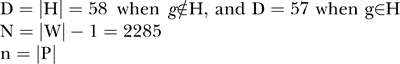
where C (a,b) is the binomial coefficient function.

To minimize the false discovery rate we used an alpha value of 0.05/2286 ≈ 2.187 e-5 (the Bonferroni correction).

### Microarray analysis.

Three independent cultures of wild-type yeast, transformed with either a plasmid that conditionally expresses OspF (without the GFP fusion) from a low-copy number plasmid (pBY011-D123-OspF) or an empty vector control plasmid (RS316), were grown overnight in selective media supplemented with 2% raffinose (raf). In the morning, each culture was diluted to an OD_600_ = 1.0 in fresh SC-URA 2% raf and incubated for 2 h at 30 °C. Galactose was then added to a final concentration of 2% to all cultures (to induce expression of OspF). After 3 h, the yeast were pelleted and snap-frozen in liquid N_2_. Total RNA was isolated using hot phenol followed by ethanol precipitation and then submitted to the Harvard Medical School Partners Healthcare Center for Genetics and Genomics (HPCGG) for further processing. HPCGG synthesize cDNA using the GeneChip Expression 3′-Amplification Reagents One-Cycle cDNA Synthesis Kit. They then preformed in vitro transcription (IVT) using the Affymetrix GeneChip Expression Amplification Reagents Kit and quantified the IVT samples with a Bio-Tek UV plate reader. Hybridization was carried out with Affymetrix yeast S98 chips according to the manual. Microarrays were scanned with a GeneChip 3000 7G Scanner controlled by the Affymetrix GCOS v1.3 operating system. We subsequently processed the raw CEL file output using the “affy” package [46] for the statistical program R [47]. Expression measurements were background corrected and normalized using the “rma” method. For each gene, an expression ratio was calculated using the (arithmetic) mean expression of the gene in each triplicate set (control and inducing).

### β-galactosidase assays.

Yeast transformed with GFP-OspF (BYO11-D123-OspF) or GFP (pRS313-GAL10-GFP) plus a RLM1-regulated *LacZ* reporter plasmid (p1434) [24] were grown overnight in selective media supplemented with 2% raf at 30 °C. In the morning, cultures were back diluted to OD_600_ = 1.0, grown at ∼23 °C, and allowed to recover for 2 h before the addition of 2% galactose. Half of the cultures were then transferred to 39 °C for 1 h, the other half kept at ∼23 °C. β-galactosidase assays were conducted as previously described [48].

### Yeast MAPK phosphorylation assays.

Yeast transformed with pRS316-GAL10-GFP-OspF or pRS316 were grown overnight in selective media supplemented with 2% raf (plus 1M sorbitol for hypoosmotic shock). In the morning, cultures were diluted (OD_600_ = 1.0) in fresh media and incubated for 2 h at 30 °C (25 °C for the heat-shock experiment). OspF was induced by addition of 2% galactose. Cells were incubated for 2 h at 30 °C (or 25 °C) before introducing the stresses. Heat shock was performed as previously described [49]. Cells were shocked by dilution 1:1 with media pre-warmed to 55 °C followed by incubation at 39 °C for 30 min. The shock response was terminated by an additional 1:1 dilution with ice-cold stop mix. The mating and invasive growth MAPK pathways were induced by the addition of 200 nM α-factor for 15 min. The HOG pathway was induced by the addition of 400 mM NaCl for 5 min. Hypoosmotic shock was performed by diluting cells 1:10 in water. In all cases, yeast were pelleted and snap-frozen at the completion of the shock procedure. Protein was isolated from yeast and subjected to SDS-PAGE. Gels were blotted to nitrocellulose and probed with the indicated antibodies according to the manufacturer's directions. The phospho-p42/44 antibody recognizes yeast phosphorylated SLT2, FUS3, and KSS1. The phospho-p38 antibody recognizes yeast phosphorylated HOG1. The PSTAIRE antibody was purchased from Santa Cruz Biotechnology, Santa Cruz, California, United States.

### Mammalian MAPK assays.

HeLa cells seeded in 6-well plates (2 ×10^5^ cells/well) were serum-starved overnight. HeLa cells were infected with *Shigella* in mid-exponential growth phase at an MOI of 10:1. Once the bacteria were added, the plates were spun at 1,000 rpm for 5 min at RT. Each of the *Shigella* strains used carries the plasmid pIL22 that constitutively expresses the afimbrial adhesin from uropathogenic E. coli to synchronize infections [50]. The 6-well plates were subsequently incubated at 37 °C for 1 h. Cells were washed with ice-cold PBS plus 1 mM Na_3_VO_4_ and 10 mM NaF and then lysed with 300 μl RIPA buffer plus protease inhibitors. Equal volumes of samples were subjected to SDS-PAGE. Gels were blotted to nitrocellulose and probed with the indicated antibodies according to the manufacturer's recommendations (Cell Signaling, http://www.cellsignal.com).

### Mouse lung infections.

C57BL/6 mice, aged 6–8 wk, were obtained from Jackson ImmunoResearch Laboratories and housed in specific pathogen-free animal facilities. The experimental procedures used in this study were approved by IACUC committee at HMS. For infection, mice were anesthetized by intramuscular injection of ketamine (12 mg/mL; Webster Veterinary Supply, Incorporated) and xylazine (4 mg/mL; Webster Veterinary Supply, Incorporated) in phosphate-buffered saline (PBS). The inoculum used for each bacterial strain was ∼ 5 ×10^7^ cfu re-suspended in PBS. Mice were inoculated intranasally in a single application of 20 μL with all mice receiving the same inoculum as determined by OD measurement and dilution plating of the inoculum. At the each time point following infection, mice were sacrificed and the lungs removed. Lungs were then fixed in 10% neutral buffered formalin and embedded in paraffin for hematoxylin and eosin staining. Images were obtained with 10× objective.

## Supporting Information

Figure S1 Yeast Growth Due to OspF Expression under Variable Conditions Each box-and-whisker plot summarizes the OD600 measurements of 11 independent yeast cultures expressing the indicated bacterial protein at t = 48 h (Protocols S1 and S3). Empty refers to yeast that carry empty vector. The boxes enclose (approximately) one quartile either side of the median. The whiskers delimit the ∼95% confidence interval for the mean (using default rendering parameters in the statistical computing software package R [47].
(210 KB JPG)

Figure S2 Subcellular Localization Patterns of GFP-OspF in Yeast and HeLa Cells (A) Shows HeLa cells transfected with C1-eGFP-OspF, a plasmid that constitutively expresses GFP-OspF from the CMV promotor. Cells were visualized ∼18 h after electroporation.
(B) Shows yeast expressing GFP-OspF from a low-copy plasmid. The picture was taken 3 h after induction of expression of GFP-OspF. Both pictures were taken at 60× magnification with a Nikon 2000 microscope equipped with a Cool Snap camera.
(65 KB TIF)

Figure S3 Spot Images of All 160 Strains Quantitatively Identified as Hypersensitive Each row contains four sets of eight spots, left to right. Each octet is from one screen: GFP (1), GFP (2), GFP-OspF (1), GFP-OspF (2), from left to right. Within each octet the left four spots are from repressing conditions (growth on glucose). The right four spots are from inducing conditions (galactose).
(4.7 MB JPG)

Figure S4 Schematic Representation of Two Analyses of Hypersensitive Null Alleles (Actual genes and ontologies are not represented.) Smooth-edge circles represent mutant yeast alleles. Lines represent SL interactions (between genes) and analogous hypersensitivity interactions of null alleles to OspF expression.
(A) Represents genes with common ontology annotations in different colors. If each colored set represents the exhaustive set of genes (in the sample space) with a given ontology annotation, and the depicted set is exhaustive of those hypersensitive to OspF, then among a sample space of thousands of genes the depicted proportions of each set hypersensitve to OspF would be highly statistically significant by the hypergeometric probability distribution.
(B) Represents congruency between a yeast mutant allele x and OspF as defined by the same kind of statistical enrichment in a different property: shared SL interaction partners. For example, given a sample space of 2,286 genes the probability of a sample of 25 genes containing seven or more of the ten genes SL to gene x is 8.8e −13. Notice that the same gene can play different roles in the two analyses.
(31 KB PDF)

Figure S5 MvA Plot of Levels of RLM1-Regulated Genes in the Presence and Absence of OspF These plots show the relationship between changes in expression level and total expression for 24 RLM1-regulated genes 3 h after the induction of expression of OspF. Changes in gene expression level are defined to be log2 (expression level with OspF/expression level without OspF). Total expression is defined as the geometric mean of the expression levels with and without OspF, i.e., square root (expression with OspF X expression without OspF). Genes normally upregulated by RLM1 are marked in green while those normally downregulated by RLM1 are marked in red. These data were obtained by averaging the expression levels observed in three parallel experiments (OspF versus empty vector).
(8 KB PDF)

Figure S6 Alignment of Protein Homologs of Shigella flexneri OspF The protein sequences of SpvC from *Salmonella enterica,* VirA from *Chromobacterium violaceum,* and HopAI1 from Pseudomonas syringae were aligned using CLUSTAL W with default parameters (http://www.ebi.ac.uk/clustalw).
(888 KB JPG)

Figure S7 Histograms of Scaled Spot Opacities from All Four Screens Gaussian curves defining the healthy colonies (spots) in each condition are shown in blue. Mixture models (sums of Gaussians) were fit to the GFP-OspF histograms to more accurately capture the center and dispersion of the healthy population. The red dashed lines indicate the Gaussian that accounts for the negative skew. The lower mode (at zero) of the first GFP screen is truncated in the figure. 3,106 spots were absent in repressing conditions and 3,156 were absent in inducing conditions.
(625 KB JPG)

Protocol S1 96-Well Yeast Liquid Growth Assays (27 KB DOC)

Protocol S2 96-Well Yeast Transformations (32 KB DOC)

Protocol S3 Analysis of Yeast Strains in Hypersensitivity Screens (68 KB DOC)

Table S1 Yeast Genes Deleted in Strains Hypersensitive to GFP-OspF Expression Descriptions are taken from the *Saccharomyces* Genome Database (http://www.yeastgenome.org).
(38 KB XLS)

Table S2 Gene Ontologies Enriched among Genes Deleted from the 83 Mutant Yeast Strains Hypersensitive to GFP-OspF Expression This table was copied directly from the output of http://llama.med.harvard.edu/cgi/func/funcassociate. Ontology names were abbreviated to fit. Ontologies that we judged too broad to be informative are indicated with gray background. Ontologies that were redundant by virtue of subset relationships are indicated by yellow background. The gene counts reflect the state of annotation in the FuncAssociate database at the time of our query.
(27 KB DOC)

Table S3 The Subset of 122 Haploid Deletion Strains Untested Due to Insufficient Transformant Replicants Dubious ORFs are shown in blue, and ORFs that encode ribosomal proteins are red.
(6 KB DOC)

Table S4 Dubious ORFs Deletion of Which Resulted in Hypersensitivity to GFP-OspF (11 KB DOC)

## Abbreviations

CWI: cell wall integrity
GFP: green fluorescent protein
MAPK: mitogen-activated protein kinase
ORF: open reading frame
SL: synthetic lethal
