# Supplementary material for: Yeast Functional Genomic Screens Lead to Identification of a Role for a Bacterial Effector in Innate Immunity Regulation
Source: PLoS Pathog. 2007 Feb 16;3(2):e21. doi: 10.1371/journal.ppat.0030021 (PMC1797620; doi:10.1371/journal.ppat.0030021)
Supplement: Supplementary file 1 [file ppat.0030021.sg004.pdf]

A

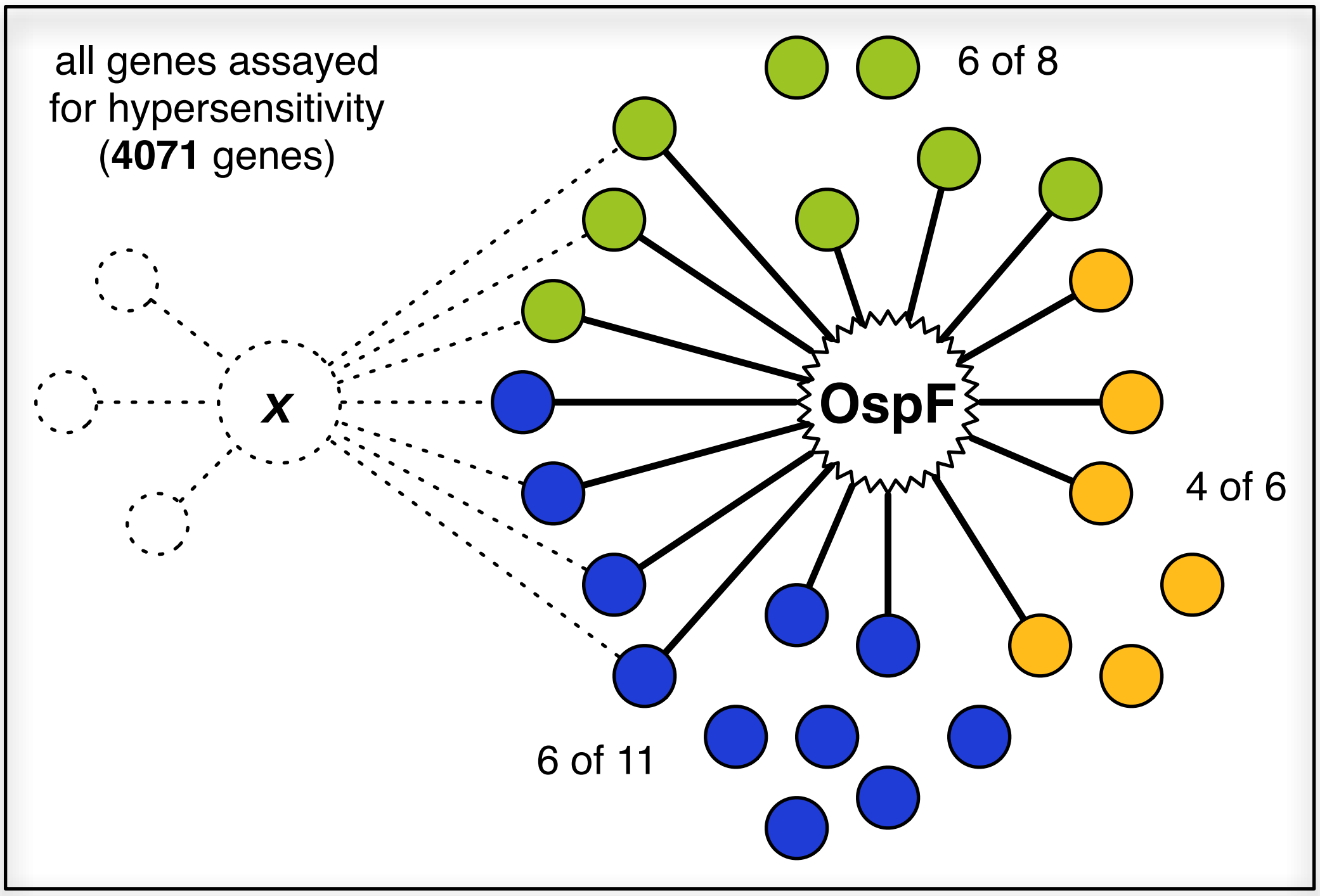
all genes assayed
for hypersensitivity
(**4071** genes)
6 of 8
x
OspF
4 of 6
6 of 11

B

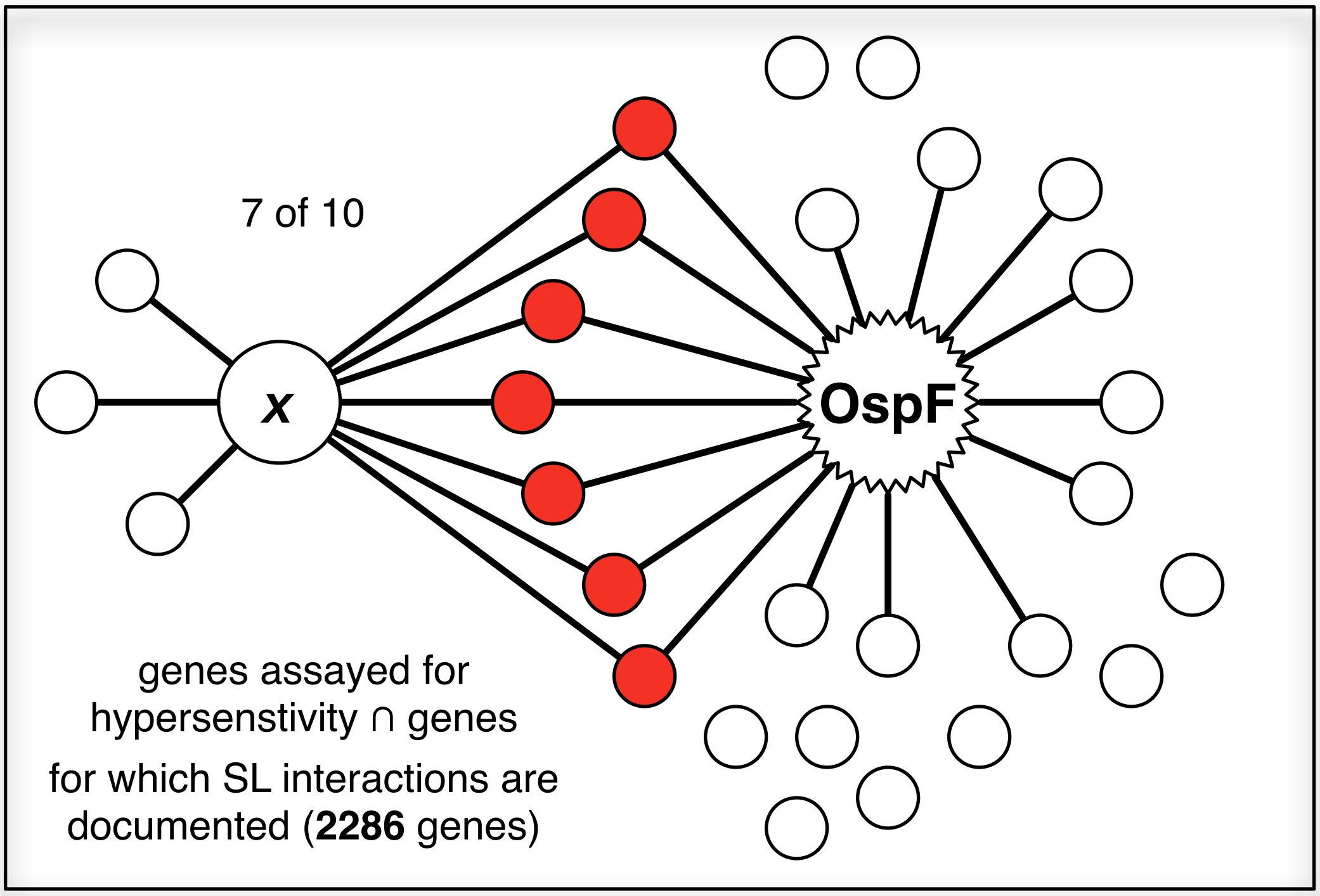
7 of 10
x
OspF
genes assayed for
hypersenstivity ∩ genes
for which SL interactions are
documented (**2286** genes)
