# Supplementary material for: Yeast Functional Genomic Screens Lead to Identification of a Role for a Bacterial Effector in Innate Immunity Regulation
Source: PLoS Pathog. 2007 Feb 16;3(2):e21. doi: 10.1371/journal.ppat.0030021 (PMC1797620; doi:10.1371/journal.ppat.0030021)
Supplement: Supplementary file 2 [file ppat.0030021.sg005.pdf]

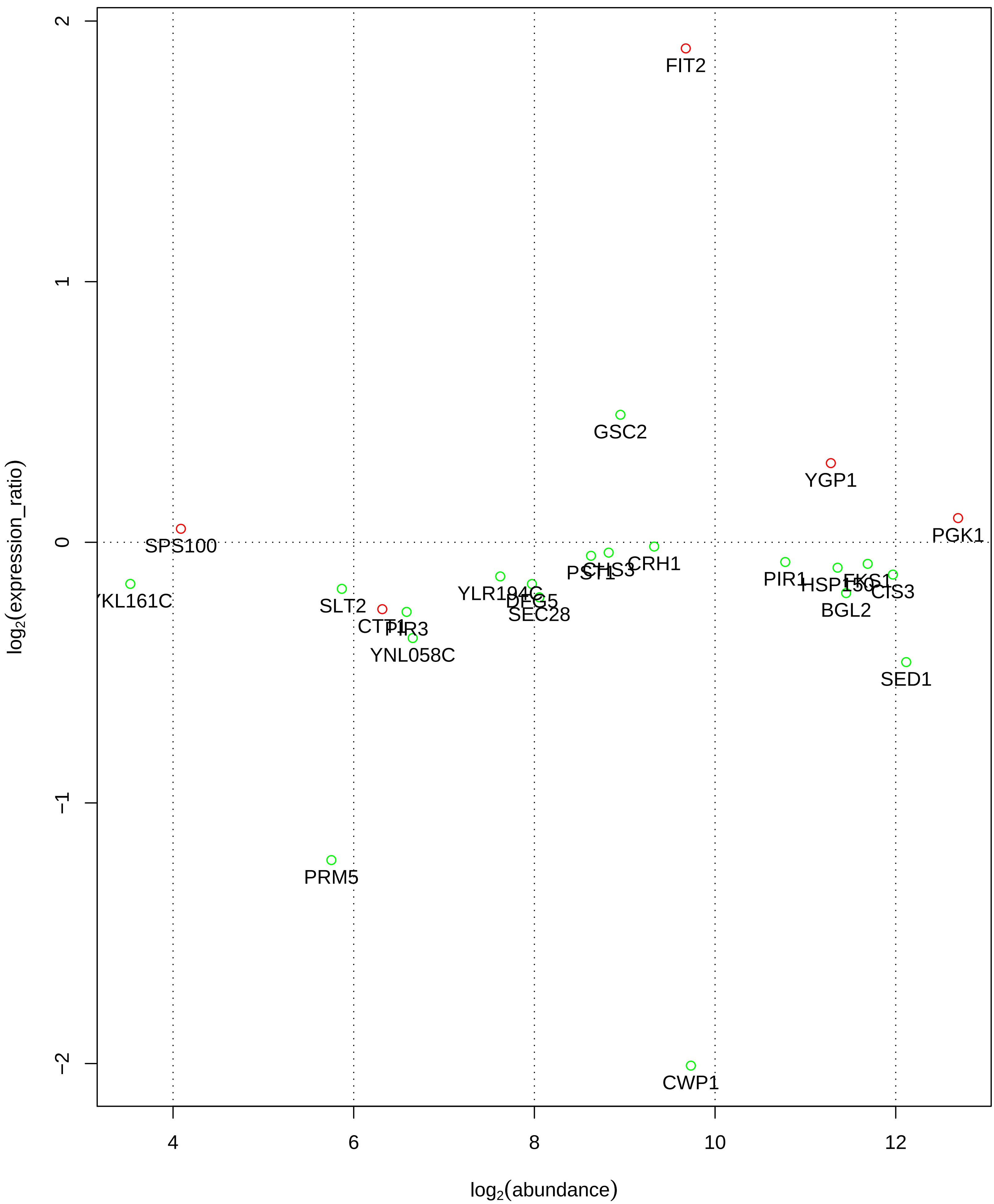

FIT2
GSC2
YGP1
PGK1
SPS100
PST1
CHS3
CRH1
PIR1
HSP150
FKS1
CIS3
BGL2
YKL161C
SLT2
CTT1
PIR3
YNL058C
YLR194C
DFG5
SEC28
SED1
PRM5
CWP1
2
1
0
−1
−2
4
6
8
10
12
log2(expression_ratio)
log2(abundance)
