# Supplementary material for: Yeast Functional Genomic Screens Lead to Identification of a Role for a Bacterial Effector in Innate Immunity Regulation
Source: PLoS Pathog. 2007 Feb 16;3(2):e21. doi: 10.1371/journal.ppat.0030021 (PMC1797620; doi:10.1371/journal.ppat.0030021)
Supplement: Supplementary file 3 [file ppat.0030021.sd001.doc]

# Protocol S1: 96-well yeast liquid growth assays

Individual yeast transformants carrying the designated plasmids were inoculated into single wells of a 96-well plate containing non-inducing selective synthetic media supplemented with 2% glucose. Plates were incubated 12-18 hours. Yeast were then transferred to a solid media tray containing non-inducing selective synthetic media supplemented with 4% glucose with a 3.18 mm diameter 96 floating pin tool (V&P Scientific, Inc., San Diego, CA). After one to two days, the yeast spots on the solid plates were transferred to fresh non-inducing liquid media containing 96-well plates using a 1.58 mm diameter 96 floating pin tool and incubated 16-18 hours such that yeast grew to OD_600_ between 0.3-0.5. The liquid cultures were then transferred with a 1.58 mm diameter floating pin tool into liquid 96-well plates containing inducing selective synthetic media (2% galactose) or inducing rich media ((YEP plus clonNAT (70 (g/ml) (Werner BioAgents, Jena, Germany) plus 2% galactose)). Plates were incubated for 48 hours without agitation and growth was measured by OD_600_ readings using a Victor II Plate Reader (Perkin Elmer, Wellesley, MA). All incubations were done at 30°C.
