# Supplementary material for: Yeast Functional Genomic Screens Lead to Identification of a Role for a Bacterial Effector in Innate Immunity Regulation
Source: PLoS Pathog. 2007 Feb 16;3(2):e21. doi: 10.1371/journal.ppat.0030021 (PMC1797620; doi:10.1371/journal.ppat.0030021)
Supplement: Supplementary file 4 [file ppat.0030021.sd002.doc]

**Protocol S2: 96-well yeast transformations**

Glycerol stocks of the yeast **MATα** haploid deletion strain collection (Open Biosystems, Huntsville, AL) were thawed and pinned onto solid YEPD plates using a 3.18 mm diameter 96 floating pin tool. Before each transformation, a 3.18 mm 96 floating pin tool was used to transfer yeast from the solid media plates to 96-well round bottom plates containing 190 μl/well of YPAD media. Plates were incubated overnight at 30°C. In the morning, the yeast were resuspended in pre-warmed 2x YPAD media (100 μl /well) and the plates were incubated at 30°C on platform shaker at 250-300 rpm for 1.5 hours. The yeast were then pelleted and resuspended in 12 μl of mix A (0.75μl 1M Tris pH 8.0, 0.15 μl 0.5M EDTA, 5 μl 10 mg/ml boiled salmon sperm DNA , ~ 200ng plasmid DNA and water). Once resuspended, 80 μl of mix B (50% PEG3350 + 8.5 μl 1M LiOAc) was immediately added to each well. Plates were incubated at 42°C for 105 minutes, the yeast were pelleted and the bulk of the transformation mix was removed. The pellets were resuspended in the residual volume and each well of the 96-well plates was spotted in quadruplicate onto a solid media tray containing synthetic complement minimal media lacking uracil (SC-URA) plus 2% glucose. A 1.58 mm 96 floating pin tool was used for this procedure.
