# Supplementary material for: Yeast Functional Genomic Screens Lead to Identification of a Role for a Bacterial Effector in Innate Immunity Regulation
Source: PLoS Pathog. 2007 Feb 16;3(2):e21. doi: 10.1371/journal.ppat.0030021 (PMC1797620; doi:10.1371/journal.ppat.0030021)
Supplement: Supplementary file 5 [file ppat.0030021.sd003.doc]

**Protocol S3: Analysis of Yeast Strains in Hypersensitivity Screens**

Colony growth was quantified by measuring spot opacity. Other researchers have used spot size, but we found that (in our protocol) spot size is too strongly affected by experimental artifacts such as non-uniform culture transfer and plate position. Edge spots, for example, are often visibly larger than interior spots. Opacity is easier to interpret and is analogous to the “optical density” measurements often used for liquid cultures.

Culture plates were scanned at 300DPI on an Epson 3200 flatbed scanner ~48 hours after inoculation (induction in the case of inoculation onto galactose containing media). At this time most healthy strains had grown to a saturation point defined by a relatively uniform spot "lawn." The resulting imagery constituted our primary data.

All images were converted to 8-bit grayscale for quantification (i.e. pixels had values in [0,255]). Earlier screens were scanned as gray-scale imagery since our quantification approach assumes one-channel imagery. Later screens were scanned in color in case a means to use full color information became available. For the purpose of quantification color images were converted to gray scale by simply extracting their red channels since investigation revealed that the red channel had the greatest range in our plates.

Spots were quantified from digital imagery using custom software developed in our lab. The software is fully automated, so no human interaction is involved in growth quantification and objectivity is insured. It partitions each plate image into a 16x24 grid of equal-sized squares such that each square contains one spot (implicitly identifying the spot's logical coordinates in the process). It then classifies the pixels of each grid square into foreground (containing culture) and background using k-means clustering. Opacity was defined as the difference in mean pixel intensities between foreground and background pixels. We used the t-test to establish the absence of a spot. Squares in which the foreground did not exceed background by 25 with a p-value  0.05 were judged to be empty and the (missing) spot's opacity value was clamped to 0. The threshold value of 25 was chosen to match visual perception of absence.

To attenuate differences in plate ranges (revealed by boxplots of foreground and background levels grouped by plate) each spot's raw opacity value was divided by the maximum opacity of all spots on the same plate. Opacity scores were thereby rescaled to the unit interval [0,1].

We normalized opacities to the healthy majority in each condition. The growth of the vast majority of colonies in all conditions was visually indistinguishable from the growth of wild-type. This majority clearly corresponds to the dominant mode in each condition's histogram (Figure S7), and opacities in the vicinity of this mode were very nearly normally distributed around it. (The second mode always appears as a sharp spike at zero because our method for detecting the absence of a spot results in all opacities near zero being mapped to zero.) We estimated the mean and standard deviation of the healthy population by fitting Gaussian curves to the histograms (minimizing squared error using the “optim” function in R). In order to account for the negative skew and more accurately capture the center and dispersion of the the healthy subpopulation we used a mixture models defined by sums of Gaussian curves for the GFP-OspF screens. We then used the parameters of the healthy population (corresponding to the blue curves in Figure S7) to normalize opacity scores in the standard way:

$$\text{opacity}_{\text{normalized}}=\frac{\text{opacity}_{\text{scaled}}-m_{\text{healthy}}}{s_{\text{healthy}}}$$

...where $m_{\text{healthy}}$ is the mean opacity of the healthy colonies, and $s_{\text{healthy}}$ is the standard deviation of opacity of healthy colonies. Under this transformation all spots for which $\left| \text{opacity}_{\text{normalized}} \right|<3$ were considered healthy. Hereafter, all references to opacity refer to normalized opacity.

**Identification of hypersensitive colonies**

Our goal was to identify growth defects that were sufficiently severe to be attributable to an interaction between the cellular perturbation caused by expression of a heterologous protein (GFP or GFP-OspF) and the perturbation caused by a gene deletion. Given the various sources of both biological and technical noise of which we were aware and the limited number of replicates we could afford to obtain we didn't believe quantitative measures of growth would be accurate enough to serve in ratio-based definitions of fitness. Thus, we confined our interest to the severest phenotypes, and we used our quantitative results primarily to obviate examination of all ~150,000 individual spots.

Since GFP-OspF has negligible effect on wild-type yeast growth (Figure S1), we simply looked for pronounced changes in growth between inducing and non-inducing media defined by:

$$D\text{opacity}=\text{opacity}_{\text{induced}}-\text{opacity}_{\text{repressed}}\text{<-}2\sqrt{2}$$

In the second OspF screen, for example, this yielded 1401 spots corresponding to between 351 and 1401 distinct strains, depending on how many of the spots were in replicates.

**Identification of hypersensitive deletion strains**

In order to identify strains, as opposed to colonies/spots, specifically hypersensitive to GFP-OspF we combined the replicates within and between four separate screens. As described above, two complete screens of the haploid deletion collection were conducted with each plasmid. Each strain in the library is represented by four spots in each screen (because of our “quad-spotting” protocol), so each strain was represented by a total of 8 biological replicates in our study.

Strains were categorized as hypersensitive if in both OspF screens at least 2 of the 4 replicate spots satisfied the condition $D\text{opacity}\text{<-}2\sqrt{2}$, and no more than 1 spot satisfied the same condition in the second GFP screen. The first GFP screen was excluded from the quantitative analysis because the transformation rate was low, but it was nonetheless included in our visual verification. The GFP-only controls were intended to identify strains for which poor growth was due primarily to galactose, not OspF expression.

Strains that did not grow in non-inducing conditions because of transformation failure obviously cannot have exhibited a significant decrease in their opacity scores in inducing conditions, so they are excluded implicitly. 122 strains (Table S3) were effectively untested because fewer than two spots grew in non-inducing conditions in one or both OspF screens (presumably due to transformation failure). Seven of these strains were deleted in dubious ORFs and 3 in ORFs encoding ribosomal proteins.

This procedure yielded 160 strains hypersensitive to OspF.

**Visual confirmation of hypersensitivity of colonies**

Using custom software developed in our lab composite images were created that combined the 5120 spot images of the 160 OspF-hypersensitive strains from all screens in both inducing and non-inducing conditions (Figure S3). (5120 = 160 strains x 2 heterologous proteins x 2 screen replicates x 2 conditions per screen x 4 spot replicates). These composite images provided for visual verification of the results of the statistical analysis. Several deletion strains (e.g. *Δfks1* and *Δsmi1*) exhibited distinctly poor growth even in glucose (Figure S3). In some cases we attributed this to incomplete repression of the *GAL* promoter by glucose [51] and counted them as true positives rather than exclude these strains as control failures.

We believe non-uniformity of a spot is related more to non-uniform culture deposition than to biological effects. Questionable spots were judged by looking for residual papillations that clearly indicate a lag in the strain's growth. See, for example, the contrast between *Δcys4* and *Δchs5* (Figure S3).

On the basis of visual examination we excluded 62 strains as false positives.

**Prioritization of strains for analysis**

Additional strains from the remaining 98 were excluded from further analysis for a variety of reasons. Ten of the genes encode ribosomal proteins. We did not expect ribosomal genes to be informative because they are pleiotropic. Seven strains were deleted in dubious ORFs (Table S4) six of which overlapped verified (protein-coding) genes on the complementary strand. We substituted the overlapped (non-dubious) gene for the dubious ORF in the list of hypersensitive strains since deletion of one almost certainly prevents correct expression of the other. Although, in no case did a mutant strain deleted in the overlapped ORF independently show a comparable phenotype, in at least one case (*Δvrp1*) this was likely due to contamination.

Finally, three of the hypersensitive strains were deleted in genes encoding proteins of galactose metabolism (*Δgal1*, *Δgal10*, *Δgal7*). These were expected to be hypersensitive since galactose was the sole carbon source in inducing media, so they were excluded.

Thus, a net of 15 additional strains were excluded from further consideration (-10 ribosomal, -3 galactose-sensitive, and -7 dubious ORFs, +5 substitutions). The 83 remaining (Table S1) constituted the hypersensitive strains on which enrichment tests were performed.

For the purposes of enrichment tests we removed all untested ORFs, 456 dubious ORFs and 144 ribosomal protein-encoding ORFs from the haploid deletion collection list. Thus, our sample space was reduced from 4773 (in the haploid deletion collection) to 4071.
