# Supplementary material for: Yeast Functional Genomic Screens Lead to Identification of a Role for a Bacterial Effector in Innate Immunity Regulation
Source: PLoS Pathog. 2007 Feb 16;3(2):e21. doi: 10.1371/journal.ppat.0030021 (PMC1797620; doi:10.1371/journal.ppat.0030021)
Supplement: Supplementary file 6 [file ppat.0030021.st002.doc]

Table S2. Gene Ontologies enriched among genes deleted from the 83 mutant yeast strains 
hypersensitive to GFP-OspF expression. This table was copied directly from the output of http://llama.med.harvard.edu/cgi/func/funcassociate . Ontology names were abbreviated to fit. Ontologies that we judged too broad to be informative are indicated with gray background. Ontologies that were redundant by virtue of subset relationships are indicated by yellow background. The gene counts reflect the state of annotation in FuncAssociate's database at the time of our query.

Rank  N   X     LOD    P        P-adj   GO Attribute	
1     6   11    1.782  2.6e-08  <0.001  0043162: ubiquitin-dependent protein catabolism via MVB pathway	
2     6   12    1.709  5.1e-08  <0.001  0010008: endosome membrane	
3     15  161   0.772  5.8e-07  <0.001  0007154: cell communication	
4     10  81    0.892  4.3e-06  0.001   0051301: cell division	
5     73  2701  0.555  6e-06    0.002   0009987: cellular process	
6     7   37    1.103  7.3e-06  0.002   0008092: cytoskeletal protein binding	
7     9   68    0.924  7.5e-06  0.002   0005938: cell cortex	
8     3   3     2.539  8.3e-06  0.002   0045014: negative regulation of transcription by glucose	
9     3   3     2.539  8.3e-06  0.002   0045013: neg. reg. of transcription by carbon catabolites	
10    3   3     2.539  8.3e-06  0.002   0000814: ESCRT II complex	
11    67  2367  0.474  1.1e-05  0.002   0005737: cytoplasm	
12    5   17    1.347  1.6e-05  0.003   0007121: bipolar bud site selection	
13    11  120   0.748  2.5e-05  0.007   0045229: external encapsulating structure org. and biogenesis	
14    11  120   0.748  2.5e-05  0.007   0007047: cell wall organization and biogenesis	
15    3   4     2.061  3.3e-05  0.014   0045990: regulation of transcription by carbon catabolites	
16    3   4     2.061  3.3e-05  0.014   0046015: regulation of transcription by glucose	
17    7   46    0.990  3.3e-05  0.014   0019954: asexual reproduction	
18    7   46    0.990  3.3e-05  0.014   0007114: cell budding	
19    8   63    0.901  3.4e-05  0.014   0015629: actin cytoskeleton	
20    8   63    0.901  3.4e-05  0.014   0019941: modification-dependent protein catabolism	
21    8   63    0.901  3.4e-05  0.014   0006511: ubiquitin-dependent protein catabolism	
22    10  102   0.778  3.4e-05  0.014   0005856: cytoskeleton	
23    8   65    0.885  4.3e-05  0.015   0000910: cytokinesis	
24    15  245   0.565  0.0001   0.035   0005515: protein binding/protein amino acid binding	
25    28  684   0.414  0.00012  0.038   0016043: cell organization and biogenesis
