# Supplementary material for: Yeast Functional Genomic Screens Lead to Identification of a Role for a Bacterial Effector in Innate Immunity Regulation
Source: PLoS Pathog. 2007 Feb 16;3(2):e21. doi: 10.1371/journal.ppat.0030021 (PMC1797620; doi:10.1371/journal.ppat.0030021)
Supplement: Supplementary file 7 [file ppat.0030021.st003.doc]

Table S3. The subset of 122 haploid deletion strains untested due to insufficient transformant 
replicants. Dubious ORFs are shown in blue, and ORFs that encode ribosomal proteins are red.

YAL010C, YAL016W, YAL047C, YBL008W, YBL047C, YBL058W, YBL099W, YBL100C, YBR021W, YBR248C, YCL030C, YCL061C, YCR015C, YDL085C-A, YDR034C-A, YDR120C, YDR300C, YDR369C, YDR388W, YDR409W, YDR414C, YEL045C, YEL046C, YER055C, YER074W-A, YER078W-A, YER086W, YFR011C, YFR016C, YFR025C, YGL007C-A, YGL028C, YGL032C, YGL033W, YGL036W, YGL037C, YGL042C, YGL050W, YGL054C, YGL058W, YGL066W, YGL072C, YGL076C, YGL095C, YGL258W-A, YGR006W, YGR051C, YGR167W, YGR208W, YGR209C, YGR215W, YGR240C-A, YHL020C, YHR004C, YHR026W, YHR041C, YHR072W-A, YHR117W, YHR135C, YHR195W, YHR198C, YIL020C, YIL039W, YIL116W, YIL149C, YIL166C, YJL056C, YJL205C, YJR104C, YJR121W, YJR130C, YJR149W, YKL026C, YKL040C, YKL057C, YKL066W, YKL069W, YKL114C, YKL138C-A, YKL146W, YKL155C, YKR016W, YKR023W, YLR131C, YLR138W, YLR242C, YLR304C, YLR381W, YLR384C, YLR399C, YML095C-A, YML112W, YMR074C, YMR091C, YMR111C, YMR114C, YNL025C, YNL055C, YNL123W, YNL138W, YNL243W, YNL252C, YNL268W, YNL273W, YNL288W, YNR059W, YOL059W, YOL108C, YOL143C, YOR061W, YOR090C, YOR091W, YOR184W, YOR240W, YOR331C, YPL129W, YPL170W, YPL198W, YPL216W, YPL222W, YPL254W, YPR036W
