# Supplementary material for: Yeast Functional Genomic Screens Lead to Identification of a Role for a Bacterial Effector in Innate Immunity Regulation
Source: PLoS Pathog. 2007 Feb 16;3(2):e21. doi: 10.1371/journal.ppat.0030021 (PMC1797620; doi:10.1371/journal.ppat.0030021)
Supplement: Supplementary file 8 [file ppat.0030021.st004.doc]

Table S4. Dubious ORFs deletion of which resulted in hypersensitivity to GFP-OspF.


Dubious ORF	Overlapped ORF	
YLR338W	YLR337C/VRP1	
YER038W-A/FMP49	YER039C/HVG1	
YDR433W	YDR432W/NPL3	
YOR135C	YOR136W/IDH2	
YNL228W	YNL227C/JJJ1	
YDR417C	YDR418W/RPL12B	
YLR111W	none
